# Addition of Fibroblast-Stromal Cell Markers to Immune Synovium Pathotypes Better Predicts Radiographic Progression at 1 Year in Active Rheumatoid Arthritis

**DOI:** 10.3389/fimmu.2021.778480

**Published:** 2021-11-23

**Authors:** Xue-Pei Zhang, Jian-Da Ma, Ying-Qian Mo, Jun Jing, Dong-Hui Zheng, Le-Feng Chen, Tao Wu, Chu-Tao Chen, Qian Zhang, Yao-Yao Zou, Jian-Zi Lin, Yan-Hui Xu, Yao-Wei Zou, Ze-Hong Yang, Li Ling, Pierre Miossec, Lie Dai

**Affiliations:** ^1^ Department of Rheumatology, Sun Yat-Sen Memorial Hospital, Sun Yat-Sen University, Guangzhou, China; ^2^ Department of Radiology, Sun Yat-Sen Memorial Hospital, Sun Yat-Sen University, Guangzhou, China; ^3^ Department of Medical Statistics, School of Public Health, Sun Yat-Sen University, Guangzhou, China; ^4^ Department of Clinical Immunology and Rheumatology, Immunogenomics and Inflammation Research Unit, University of Lyon and Hospices Civils de Lyon, Lyon, France

**Keywords:** rheumatoid arthritis, synovial tissue, myeloid-stromal pathotype, stromal cells, radiographic progression

## Abstract

**Objectives:**

This study aims to investigate if addition of fibroblast-stromal cell markers to a classification of synovial pathotypes improves their predictive value on clinical outcomes in rheumatoid arthritis (RA).

**Methods:**

Active RA patients with a knee needle synovial biopsy at baseline and finished 1-year follow-up were recruited from a real-world prospective cohort. Positive staining for CD20, CD38, CD3, CD68, CD31, and CD90 were scored semiquantitatively (0-4). The primary outcome was radiographic progression defined as a minimum increase of 0.5 units of the modified total Sharp score from baseline to 1 year.

**Results:**

Among 150 recruited RA patients, 123 (82%) had qualified synovial tissue. Higher scores of CD20+ B cells, sublining CD68+ macrophages, CD31+ endothelial cells, and CD90+ fibroblasts were associated with less decrease in disease activity and greater increase in radiographic progression. A new fibroblast-based classification of synovial pathotypes giving more priority to myeloid and stromal cells classified samples as myeloid-stromal (57.7%, 71/123), lymphoid (31.7%, 39/123), and paucicellular pathotypes (10.6%, 13/123). RA patients with myeloid-stromal pathotype showed the highest rate of radiographic progression (43.7% vs. 23.1% vs. 7.7%, *p* = 0.011), together with the lowest rate of Boolean remission at 3, 6, and 12 months. Baseline synovial myeloid-stromal pathotype independently predicted radiographic progression at 1 year (adjusted OR: 3.199, 95% confidence interval (95% CI): 1.278, 8.010). Similar results were obtained in a subgroup analysis of treatment-naive RA.

**Conclusions:**

This novel fibroblast-based myeloid-stromal pathotype could predict radiographic progression at 1 year in active RA patients which may contribute to the shift of therapeutic decision in RA.

## Introduction

Rheumatoid arthritis (RA) is a highly heterogeneous disease with variable functional prognosis, level of joint destruction, and response to therapy (1). Treat-to-target strategy (T2T) targeting remission or low disease activity (LDA) is widely applied to RA treatment, resulting in better disease management and quality of life (2, 3). However, there remains a huge unmet need in RA management under the real-life situation (1). Around 20% of RA patients with sustained remission still show radiographic progression (4). Studies have been done to explore predictive biomarkers for poor prognosis, including genetic background, serologic biomarkers, and early imaging findings (5–7). Despite the theoretical advantages, simple measures such as erythrocyte sedimentation rate (ESR) and C-reactive protein (CRP) are not specific to joint disease and the predictive value seems unreliable (8).

Access to lesion is the gold standard in many diseases ranging from cancer to lupus nephritis, but this has not been applied to the synovial tissue in RA (9–11). Analysis of the synovial tissue may represent the golden criterion for the evaluation of arthropathies and could be a promising approach for better understanding of pathogenesis and prognostic prediction in RA (11–13). Histological synovitis scores, such as the Krenn synovitis score, have been used to identify inflammatory joint diseases by semiquantitative evaluation of low-grade or high-grade synovitis, and this could be helpful for the diagnosis of RA (14, 15). As the cellular composition and function in synovial tissue are the driving force of synovitis and joint destruction in RA, immunologic synovitis assessment introduced and updated synovial cellular and molecular signatures have broadened the value of synovial biopsy as a potential tool for disease classification and therapeutic outcome prediction (16, 17). Current synovial immunohistological assessments proposed by Pitzalis et al. mainly focus on the infiltrated immune cells in the synovium, to classify RA synovium into different synovial pathotypes. These have been identified to contribute to distinguish treatment response, especially in early and treatment-naive RA (17–19).

In addition to classical immunocytes, other cell types contribute to the synovitis amplification and persistence in RA (20). Neovascularization recognized by CD31 immunostaining of endothelial cells is the basis of synovial pannus formation (21). Stromal cells defined as fibroblasts or synoviocytes contribute through cell interactions with immunocytes to increase local cytokine production, increased invasion, and acquired molecular changes involved in chronicity (22, 23). Recent work has shown the great diversity of synovial fibroblasts. The CD90+ subset, also known as THY1+ subset, which is mainly expressed in the perivascular zone of the sublining area has been well investigated at the single-cell level (24, 25). Whether addition of the marker of CD90+ fibroblasts improves the value of immunohistological assessments remains unknown. In this prospective cohort study, giving priority to myeloid and stromal cells, we proposed a new classification of synovial pathotypes in RA and explored the predictive value of baseline synovial pathotypes on clinical outcomes, especially 1-year radiographic progression.

## Materials and Methods

### Study Design and Patients

Patients with RA according to the 2010 American College of Rheumatology (ACR)/European League Against Rheumatism (EULAR) classification criteria (26) were recruited between May 2010 and August 2020 from a real-world prospective cohort carried in the Department of Rheumatology, Sun Yat-Sen Memorial Hospital as we previously reported (7, 27, 28). Inclusion criteria included RA patients who had a synovial biopsy at baseline and had finished regular visits at 1 year. RA patients overlapping other autoimmune diseases were excluded (7, 27). All patients were treated according to the EULAR recommendations of the “treat-to-target” strategy. Adjustment of treatment based on shared decision-making and the therapeutic target was defined as Disease Activity Score in 28 joints with four variables including C-reactive protein (DAS28-CRP) <2.6 in all patients or <3.2 in patients with long disease duration (>24 months) as previously described (28). Ethical approval was provided by the Medical Ethics Committee of Sun Yat-Sen Memorial Hospital (SYSEC-2009-06 and SYSEC-KY-KS-012). All patient consents were obtained before biopsy and collection of clinical data.

### Data Collection

Available demographic and clinical data were collected at baseline and at 1-, 3-, 6-, and 12-month visits, as previously described (7, 27, 28). Disease activity was categorized into four states according to the Clinical Disease Activity Index (CDAI): high (>22), moderate (10~22), low (2.8~10), and remission (≤2.8). Active disease activity was defined as CDAI >2.8. X-ray examination of bilateral hands and wrists was assessed by the Sharp/van der Heijde modified score at enrollment and 12 months (7, 27, 28). The modified total Sharp score (mTSS) included subscores of joint erosion (JE) and joint space narrowing (JSN). Radiographic interruption of cortical bone was defined as bone erosion (29).

### Outcome Assessments

The primary outcome was radiographic progression defined as a minimum increase of 0.5 units in the mTSS (ΔmTSS ≥0.5) from baseline to 1 year (28). The secondary outcomes were therapeutic responses including the percentages of patients achieving ACR/EULAR Boolean remission (28TJC, 28SJC, PtGA, CRP (in mg/dl) ≤1) (30, 31), CDAI remission (CDAI ≤2.8), or LDA target (CDAI ≤10) at each visit.

### Synovial Immunohistochemistry and Synovitis Assessment

All recruited RA patients had a Parker-Pearson needle biopsy of synovium from swollen knee at baseline (27, 32). The qualified synovium was defined as at least six pieces and at least 5 fields containing intact lining layer at magnification of ×400 (27, 32). Hematoxylin and eosin (H&E) and immunohistochemical staining was performed on serial sections. Mouse monoclonal antibodies: anti-CD20 (clone L26, B cells), anti-CD38 (clone SPC32, plasma cells), anti-CD3 (clone PS1, T cells), anti-CD68 (clone KP1, macrophages), and anti-CD31 (clone OTI2F10, endothelial cells), all antibodies were purchased from Zhongshan Biotechnology Co., Ltd., Guangdong, China, except anti-CD90; fibroblast was from Abcam, Cambridge, MA, USA.

Histopathological assessments of synovitis performed on the H&E staining were graded according to the Krenn synovitis score system (15) that contains three subscores for lining hyperplasia, inflammatory infiltration, and synovial stromal activation, each of which was scored from 0 to 3. The densities of positive-staining cells for CD20, CD38, CD3, sublining CD68+ macrophages (SL-CD68), CD31, and CD90 were assessed by two independent trained investigators using a preproposed semiquantitative (0~4) scale score: 0 = no staining, 1 = <25%, 2 = 25%~50%, 3 = 50%~75%, and 4 = >75% positive staining (33). The interexaminer agreement was evaluated by intraclass correlation coefficient with mean values of 0.935~0.962.

### Statistical Analysis

According to distributions, qualitative variables were reported as frequencies and percentages, and quantitative variables were reported as mean and standard deviation (SD) or median and interquartile range (IQR). SPSS version 25.0 (IBM, Armonk, NY, USA) was used for statistical analyses.

Principal component analysis of six synovial cellular scores including CD20, CD38, CD3, SL-CD68, CD31, and CD90 was performed to investigate the intercellular connections between these six cell types. Kaiser-Meyer-Olkin test was used to evaluate sampling adequacy, while Bartlett’s test was used to determine if the correlation matrix is an identity matrix. A more easily interpretable pathological pattern was achieved by using orthogonal rotation (34).

The relationships between synovial cellular scores with clinical characteristics were evaluated by Spearman’s rank-order correlation test. The differences among two or three subgroups were compared by the Chi-square test or Fisher’s exact test for qualitative variables. t-test or Mann-Whitney U-test was used for quantitative variables between two subgroups, and analysis of variance (ANOVA) or Kruskal-Wallis test for quantitative variables among three subgroups, with Bonferroni correction for multiple comparisons. Logistic regression analyses were performed to identify the relationship between synovial pathotypes and 1-year radiographic progression with the adjustment of potential confounders at baseline. The statistical significance was set at *p-*value <0.05 in two-tailed tests.

## Results

### Baseline Characteristics of RA Patients

**Figure 1:**
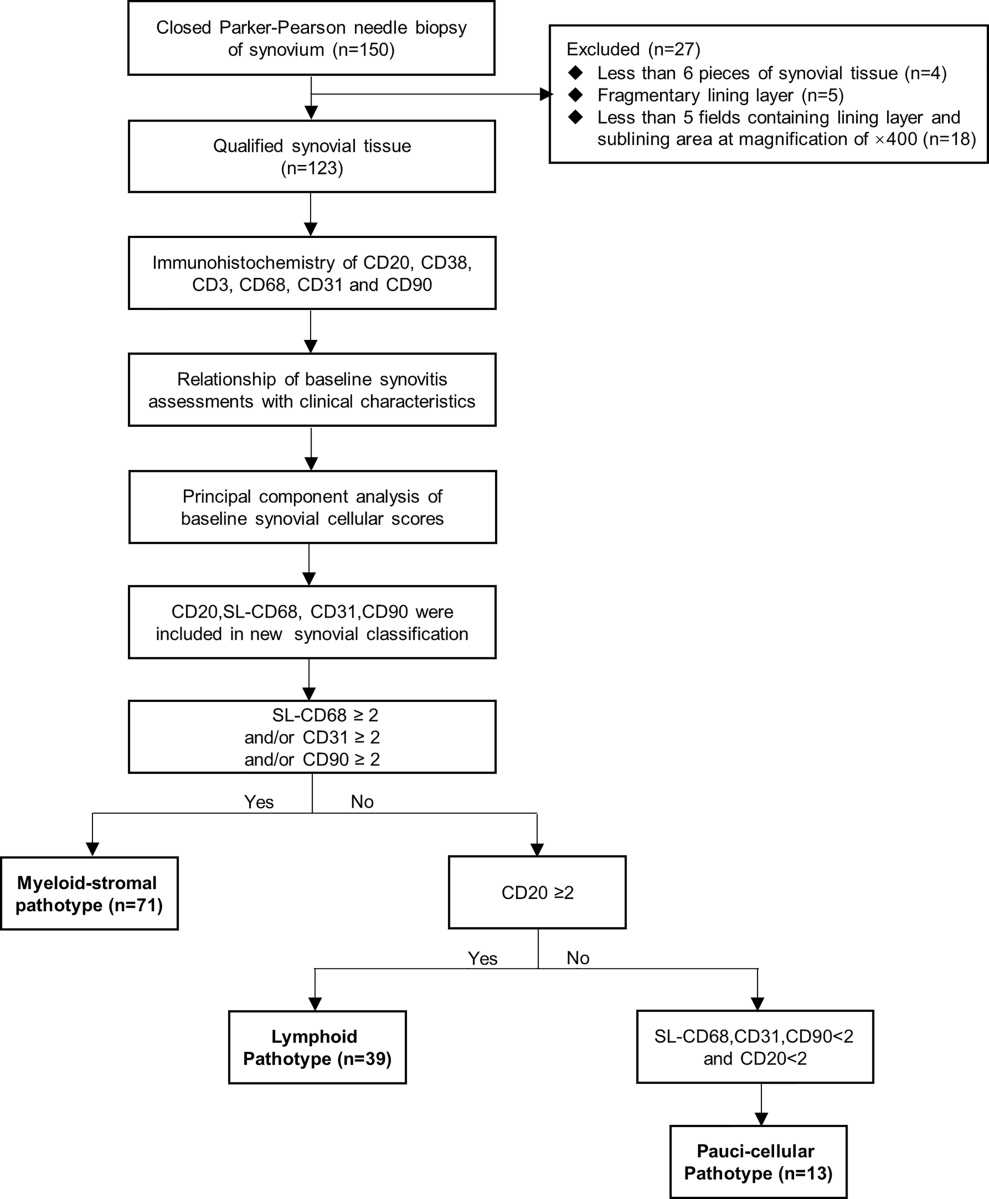
Flow diagram of study design.

Among 150 RA patients who had a synovial biopsy at baseline and finished the follow-up at 1 year, 123 (82%) had qualified synovial tissue (**Figure 1**). Their demographic and clinical features at baseline are summarized in **Table 1**. The mean age was 50 ± 14 years with 77.2% female. The mean disease duration was 58 ± 73 months, and 13.0%, 30.1%, and 47.2% of patients had disease duration of <6, <12, and >24 months, respectively. All included patients had active RA, and 64.2%, 29.3%, and 6.5% of patients had high, moderate, and low disease activity, respectively. There were 95 (77.2%) patients with bone erosions. There were 49 (39.8%) treatment-naive patients, who had not received corticosteroids or disease-modifying antirheumatic drug (DMARD) treatment for at least 6 months before recruitment.

**Table 1:** Baseline characteristics of included RA patients.

Characteristics	All patients (*n* = 123)	Treatment-naive patients (*n* = 49)
Age (year, mean ± SD)	50 ± 14	51 ± 12
Female [*n* (%)]	95 (77.2)	39 (79.6)
Disease duration (months, mean ± SD)	58 ± 73	55 ± 65
Smoking [*n* (%)]	21 (17.1)	9 (18.4)
**Core disease activity indicators**		
TJC28 [median (IQR)]	9 (4, 14)	9 (5, 16)
SJC28 [median (IQR)]	6 (2, 11)	6 (3, 11)
PtGA [median (IQR)]	6 (5, 8)	6 (5, 8)
PrGA [median (IQR)]	6 (4, 8)	6 (4, 8)
Pain VAS [median (IQR)]	5 (4, 7)	5 (4, 7)
ESR (mm/h) [median (IQR)]	69 (44, 95)	65 (44, 90)
CRP (mg/L) [median (IQR)]	30.7 (11.9, 55.1)	23.0 (9.4, 51.1)
Positive RF 9*n* (%)	99 (80.5)	39 (79.6)
Positive ACPA [*n* (%)]	102 (82.9)	42 (85.7)
DAS28-CRP [median (IQR)]	4.7 (4.0, 5.3)	4.6 (4.0, 5.1)
SDAI [median (IQR)]	31.5 (21.2, 42.5)	30.6 (22.0, 42.7)
CDAI [median (IQR)]	27 (18, 39)	28 (19, 40)
**Functional indicator**		
HAQ-DI 9median (IQR)	1.15 (0.50, 2.00)	1.13 (0.63, 1.13)
**Radiographic assessments**		
mTSS [median (IQR)]	10 (3, 37)	8 (2, 28)
JSN subscore [median (IQR)]	4 (0, 17)	2 (0, 11)
JE subscore [median (IQR)]	6 (1, 19)	5 (1, 18)
Bony erosion [*n* (%)]	95 (77.2)	38 (77.6)
**Previous medications**		
Treatment naive^Δ^ [*n* (%)]	49 (39.8)	–
Corticosteroids [*n* (%)]	59 (48.0)	–
csDMARDs [*n* (%)]	51 (41.5)	–
Biologic agents [*n* (%)]	13 (10.6)	–

ACPA, anticyclic citrullinated peptide antibody; CRP, C-reactive protein; CDAI, Clinical Disease Activity Index; DAS28-CRP, Disease Activity Score in 28 joints including CRP; ESR, erythrocyte sedimentation rate; HAQ-DI, Stanford Health Assessment Questionnaire Disability Index; JSN, Joint space narrowing; JE, joint erosion; mTSS, modified total Sharp score; PtGA, patient global assessment of disease activity; PrGA, provider global assessment of disease activity; Pain VAS, pain visual analog scale; RF, rheumatoid factor; SDAI, Simplified Disease Activity Index; SJC28, 28-joint swollen joint count; TJC28, 28-joint tender joint count. Treatment naive^Δ^ are those without previous corticosteroid or DMARD therapy for at least 6 months before enrollment.

### Therapeutic Response and Radiographic Outcome After 1-Year Follow-Up

After a 1-year follow-up, significant improvement was observed in most disease activity indicators (**Supplementary Figure S1**). There were 29.3%, 31.7%, and 34.1% of RA patients who achieved an ACR/EULAR Boolean remission at 3, 6 and 12 months, 30.1%, 35.0%, and 32.5% of RA patients achieved Clinical Disease Activity Index (CDAI) remission, and 65.9%, 71.5%, and 68.3% of RA patients achieved CDAI LDA target, respectively. A total of 41 (33.3%) RA patients showed radiographic progression and eight (6.5%) patients showed rapid radiographic progression (RRP, ΔmTSS ≥5) at 12 months.

### Relationship of Baseline Synovitis Assessments With Clinical Characteristics

**Figure 2:**
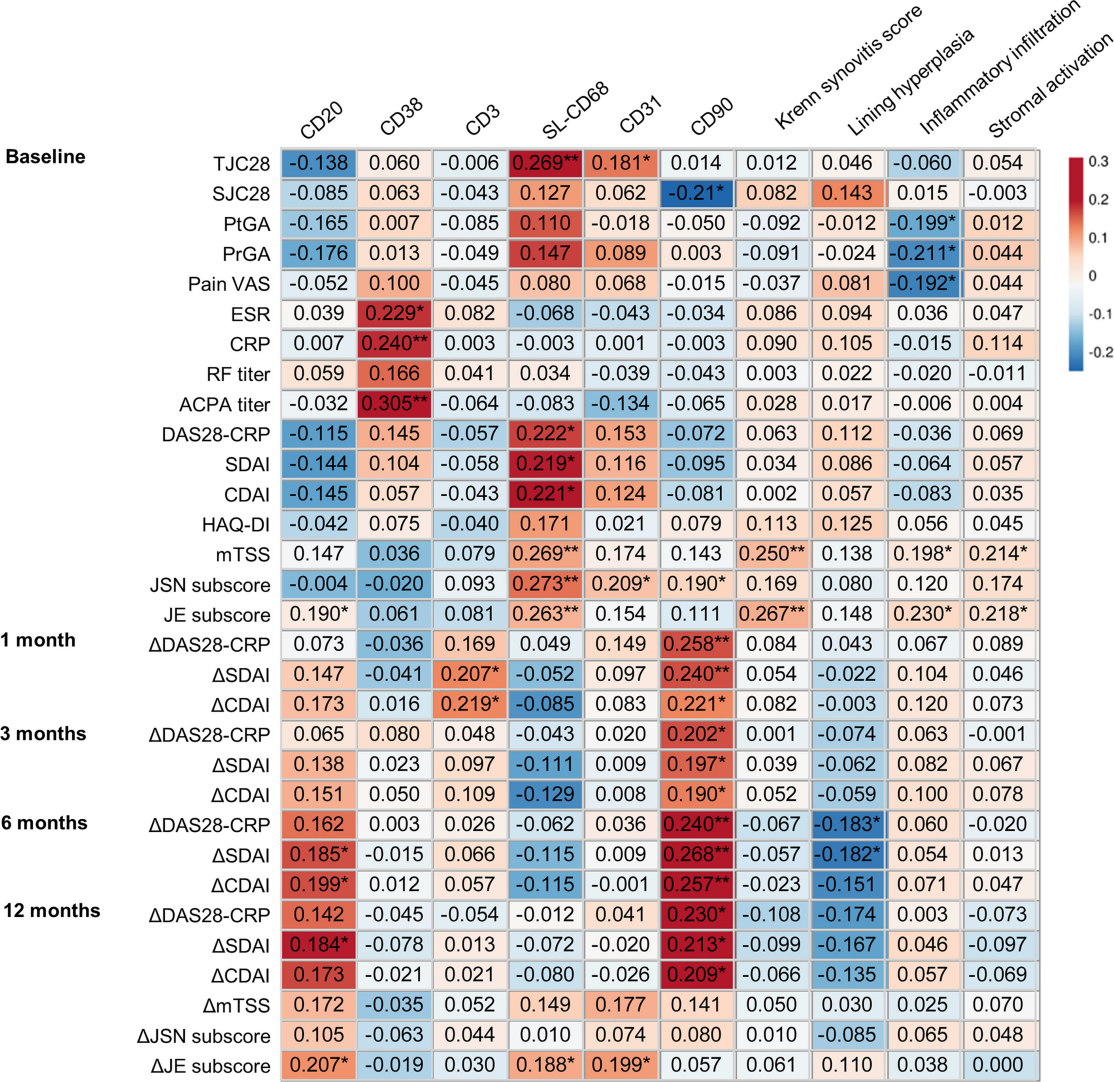
Relationship of baseline synovitis assessments with clinical characteristics of RA patients during 1-year follow up. ΔDAS28-CRP(1/3/6/12)/ΔSDAI(1/3/6/12)/ΔCDAI(1/3/6/12), changes in DAS28-CRP/SDAI/CDAI from baseline to 1/3/6/12 months; ΔmTSS(12)/ΔJSN(12)/ΔJE(12), changes in mTSS/JSN subscore/JE subscore from baseline to 12 months. ^*^ *p* < 0.05; ^**^ *p* < 0.01.

The total Krenn synovitis score and its subscores of inflammatory infiltration and stromal activation were positively associated with baseline mTSS and JE subscore (*r* = 0.198~0.230, all *p* <0.05, **Figure 2**). The Krenn synovitis score and the three subscores showed no correlation with changes of clinical indicators, except that the subscore of lining hyperplasia was positively associated with a higher decrease in Disease Activity Score in 28 joints including CRP (DAS28-CRP) and Simplified Disease Activity Index (SDAI) at 6 months (*r* = −0.183, *p* = 0.042 and *r* = −0.182, *p* = 0.044).

For the synovitis assessment of immune cell subsets, the score of CD20+ B cells was positively associated with baseline JE subscore (*r* = 0.190, *p* = 0.035, **Figure 2**). The score of SL-CD68+ macrophages was positively associated with baseline DAS28-CRP, SDAI, CDAI, mTSS, JSN subscore, and JE subscore (*r* = 0.219~0.273, all *p* < 0.05). During 1-year follow-up, the higher score of synovial CD20+ B cells at baseline was correlated with a lower decrease in SDAI, CDAI at 6 months, and in SDAI at 12 months, as well as a greater increase in JE subscore at 12 months (*r* = 0.184~0.207, all *p* < 0.05). The higher score of SL-CD68+ macrophages at baseline was associated with a greater increase in JE subscore (*r* = 0.188, *p* = 0.038).

The scores of CD38+ plasma cells and CD3+ T cells showed no correlation with baseline characteristics, therapeutic response, or radiographic outcome except for positive association of the CD38+ plasma cell score with baseline ESR, CRP, and anticyclic citrullinated peptide antibody (ACPA) titer (*r* = 0.229~0.305, all *p* < 0.05), and positive association of the CD3+ T-cell score with a less decrease in SDAI and CDAI at 1 month (*r* = 0.207, *p* =0.022 and *r* = 0.219, *p* = 0.015).

### Relationship of Baseline Synovial Stromal Cells With Clinical Characteristics

Considering the critical role of synovial stromal cells in RA pathogenesis, the assessment of CD31+ endothelial cells and CD90+ fibroblasts was performed. The scores of CD31+ endothelial cells and CD90+ fibroblasts were positively associated with baseline JSN subscore (*r* = 0.209, *p* = 0.020 and *r* = 0.190, *p* = 0.035, **Figure 2**). During 1-year follow-up, the higher score of CD90+ fibroblasts was correlated with a lower reduction in DAS28-CRP, SDAI, CDAI at 1, 3, 6, and 12 months (*r* = 0.190~0.268, all *p* < 0.05). The higher score of CD31+ endothelial cells was associated with a greater increase in JE subscore (*r* = 0.199, *p* = 0.028). These results implied that evaluation of synovial pathotypes adding stromal cell markers to the synovial immunohistological classification may provide additional valuable information.

### Establishment of a Fibroblast-Based Classification of Synovial Pathotypes

To establish a new synovial pathotype classification in a real-world cohort, the principal component analysis of baseline synovial cellular scores was performed to identify intercellular connections. Two factors were extracted by orthogonal transformations; one factor was positively related to the scores of synovial CD20+ B cells, CD38+ plasma cells, and CD3+ T cells, which were then named as lymphoid cell factor. The other factor was positively related to the scores of sublining CD68+ macrophages, CD31+ endothelial cells, and CD90+ fibroblasts, which was named as myeloid-stromal factor (**Table 2**).

**Table 2:** Matrix of rotating factor loadings for two synovial cellular patterns.

Cell types in synovium	Lymphoid factor	Myeloid-stromal factor
CD20+ B cells	0.703	−0.232
CD38+ plasma cells	0.463	−0.100
CD3+ T cells	0.773	0.289
Sublining CD68+ macrophages	−0.026	0.765
CD31+ endothelial cells	−0.255	0.748
CD90+ fibroblasts	0.051	0.684

Two factors were derived by principal component analysis after entering the six cell types in synovium into the factor procedure. The factors were rotated by orthogonal transformations.

**Figure 3:**
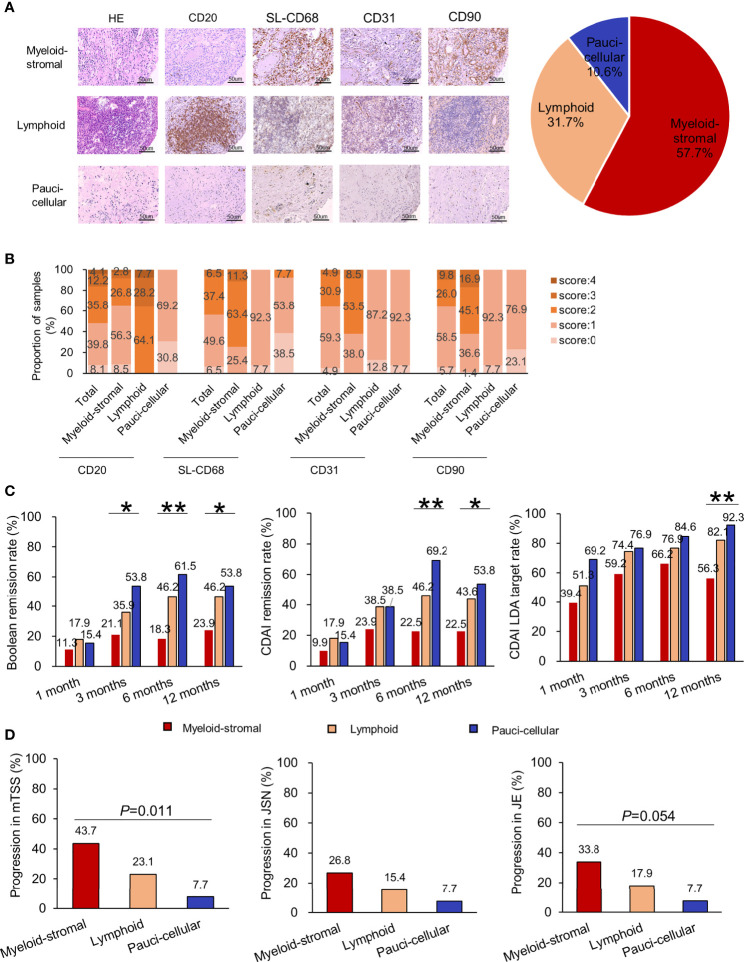
Relationship of synovial pathotypes in the new synovial pathotype classification and clinical characteristics in RA patients. **(A)** Representative H&E staining, immunohistochemistry, and distribution of synovial pathotypes. **(B)** Distribution of each synovial cellular score. **(C)** Comparisons of therapeutic responses (rates of ACR/EULAR Boolean remission, CDAI remission, CDAI LDA) among different synovial pathotypes at 1, 3, 6, and 12 months. **(D)** Comparisons of radiographic progression in mTSS, JSN subscore, and JE subscore at 1 year among different synovial pathotypes. Progression in JSN or JE, defined as a minimum increase of 0 unit in the JSN or JE subscore from baseline to 1 year. ^*^ *p* < 0.05; ^**^ *p* < 0.01 (Chi-square test or Fisher’s exact test).

Considering the importance of the scores of CD20, SL-CD68, CD31, and CD90, but not of CD3 and CD38, a new synovial pathotype classification was established. According to the 0~4 scoring system, as there were roughly half of the samples rated as <2 for CD20, SL-CD68, CD31, and CD90 (50.4%~63.4%, **Figures 3A, B** **)**, the cutoff value was then set at a score of 2. The new classification included three options: (1) Myeloid-stromal: SL-CD68 ≥2 and/or CD31 ≥2 and/or CD90 ≥2; (2) Lymphoid: CD20 ≥2; and (3) Paucicellular: SL-CD68, CD31, CD90 <2, and CD20 <2 (**Figure 1**). If synovial tissue fragments from the same patient exhibited different pathotypes, both the myeloid and stromal cells were given priority (the SL-CD68+, CD31+, and/or CD90+ cells were preferentially assessed, then CD20+ cells).

### Therapeutic Responses in RA Patients With New Synovial Pathotypes

According to the new classification, 57.7% (71/123) of RA samples were classified as synovial myeloid-stromal pathotype, 31.7% (39/123) as lymphoid pathotype, and 10.6% (13/123) as paucicellular pathotype (**Figure 3A**). Comparing their baseline characteristics, RA patients with myeloid-stromal pathotype showed higher levels of DAS28-CRP, mTSS, JSN subscore, and JE subscore (all *p* < 0.05, **Supplementary Table S1**).

During 1-year follow-up, RA patients with baseline myeloid-stromal pathotype showed the poorest therapeutic response with the lowest rate of Boolean remission (21.1% vs. 35.9% vs. 53.8%, *p* =0.032, **Figure 3C**), and the highest DAS28-CRP (2.6 (2.0, 3.6) vs. 2.1 (1.5, 3.1) vs. 2.6 (1.6, 3.1), *p* = 0.039, **Supplementary Figure S2**) at 3 months. They showed the lowest rate of Boolean remission (18.3% vs. 46.2% vs. 61.5%, *p* =0.001) and CDAI remission (22.5% vs. 46.2% vs. 69.2%, *p* = 0.001), and the highest DAS28-CRP, SDAI, CDAI, and HAQ-DI (all *p* < 0.05) at 6 months. They showed the lowest rate of Boolean remission (23.9% vs. 46.2% vs. 53.8%, *p* = 0.018), CDAI remission (22.5% vs. 43.6% vs. 53.8%, *p* = 0.017), and CDAI LDA target (56.3% vs. 82.1% vs. 92.3%, *p* = 0.003), as well as the highest DAS28-CRP, SDAI, CDAI, and HAQ-DI (all *p* < 0.05) at 12 months. There were no significant differences in initial treatment and cumulative doses of corticosteroids or DMARDs at 6 and 12 months among the three subgroups (all *p* > 0.05, **Table 3**).

**Table 3:** Medications in RA patients with three new synovial pathotypes.

Medications	Myeloid-stromal pathotype (*n* = 71)	Lymphoid pathotype (*n* = 39)	Paucicellular pathotype (*n* = 13)	*p* ^a^
**Initial medications**				
Treatment-naive^△^ [*n* (%)]	25 (35.2)	18 (46.2)	6 (46.2)	0.472
Glucocorticoids [*n* (%)]	35 (49.3)	19 (48.7)	5 (38.5)	0.767
Methotrexate [*n* (%)]	16 (22.5)	9 (23.1)	3 (23.1)	0.997
Leflunomide [*n* (%)]	16 (22.5)	6 (15.4)	2 (15.4)	0.613
Hydroxychloroquine [*n* (%)]	3 (4.2)	3 (7.7)	0 (0)	0.701
Sulfasalazine [*n* (%)]	2 (2.8)	3 (7.7)	0 (0)	0.384
Cyclosporin A [*n* (%)]	0 (0)	1 (2.6)	0 (0)	0.423
Biologic agents [(*n* (%)]	7 (9.9)	6 (15.4)	0 (0)	0.282
Tocilizumab [*n* (%)]	6 (8.5)	5 (12.8)	0 (0)	0.414
TNF-α inhibitors [*n* (%)]	1 (1.4)	1 (2.6)	0 (0)	1.000
**6-month cumulative doses of medications**				
Corticosteroids [mg, median (IQR)]	1,350 (825–1,725)	1,275 (900–1,800)	1,275 (863–1,763)	0.947
Methotrexate [mg, median (IQR))	260 (260–348)	260 (260–315)	325 (260–353)	0.138
Leflunomide [mg, median (IQR)]	1,800 (900–2,700)	1,800 (600–2,700)	1,800 (450–2,775)	0.971
Hydroxychloroquine [mg, median (5th–95th percentile range)]	0 (0–61,200)	0 (0–72,000)	0 (0–NA)	0.654
Sulfasalazine (mg, median [5th–95th percentile range)]	0 (0–270,000)	0 (0–270,000)	0 (0–NA)	0.883
Cyclosporin A (mg, median [5th–95th percentile range)]	0 (0–12,600)	0 (0–3,000)	0 (0–0)	0.557
Tocilizumab (mg, median [5th–95th percentile range)]	0 (0–1,600)	0 (0–1,600)	0 (0–NA)	0.743
TNF-α inhibitors [*n* (%)]	1 (1.4)	1 (2.6)	0 (0)	1.000
Tofacitinib [*n* (%)]	3 (4.2)	1 (2.6)	0 (0)	1.000
**1-year cumulative doses of medications**				
Corticosteroids [mg, median (IQR)]	2,175 (1,175–2,869)	1,763 (1,275–2,775)	2,025 (1,312–3,225)	0.747
Methotrexate [mg, median (IQR)]	565 (520–715)	595 (520–650)	650 (548–743)	0.209
Leflunomide [mg, median (IQR)]	3,600 (1,800–4,950)	3,600 (1,800–5,550)	3,600 (1,800–5,625)	0.675
Hydroxychloroquine [mg, median (5th–95th percentile range)]	0 (0–108,000)	0 (0–144,000)	0 (0–NA)	0.815
Sulfasalazine [mg, median (5th–95th percentile range)]	0 (0–450,000)	0 (0–540,000)	0 (0–NA)	0.594
Cyclosporin A [mg, median (5th–95th percentile range)]	0 (0–22,140)	0 (0–18,000)	0 (0–0)	0.351
Tocilizumab [(mg, median (5th–95th percentile range)]	0 (0–2,560)	0 (0–2,400)	0 (0–NA)	0.633
TNF-α inhibitors [*n* (%)]	1 (1.4)	1 (2.6)	0 (0)	1.000
Tofacitinib [*n* (%))]	3 (4.2)	1 (2.6)	0 (0)	1.000

NA, data not applicable. ^a^Comparisons of medicines among three new synovial pathotypes during 1-year follow-up by the Chi-square test or Fisher’s exact test for qualitative variables, ANOVA, or Kruskal-Wallis test for quantitative variables. TNF, tumor necrosis factor. Treatment naive^Δ^ are those without previous corticosteroid or DMARD therapy for at least 6 months before enrollment.

### Predictive Value of New Synovial Pathotypes on Radiographic Progression at 1 Year

**Figure 4:**
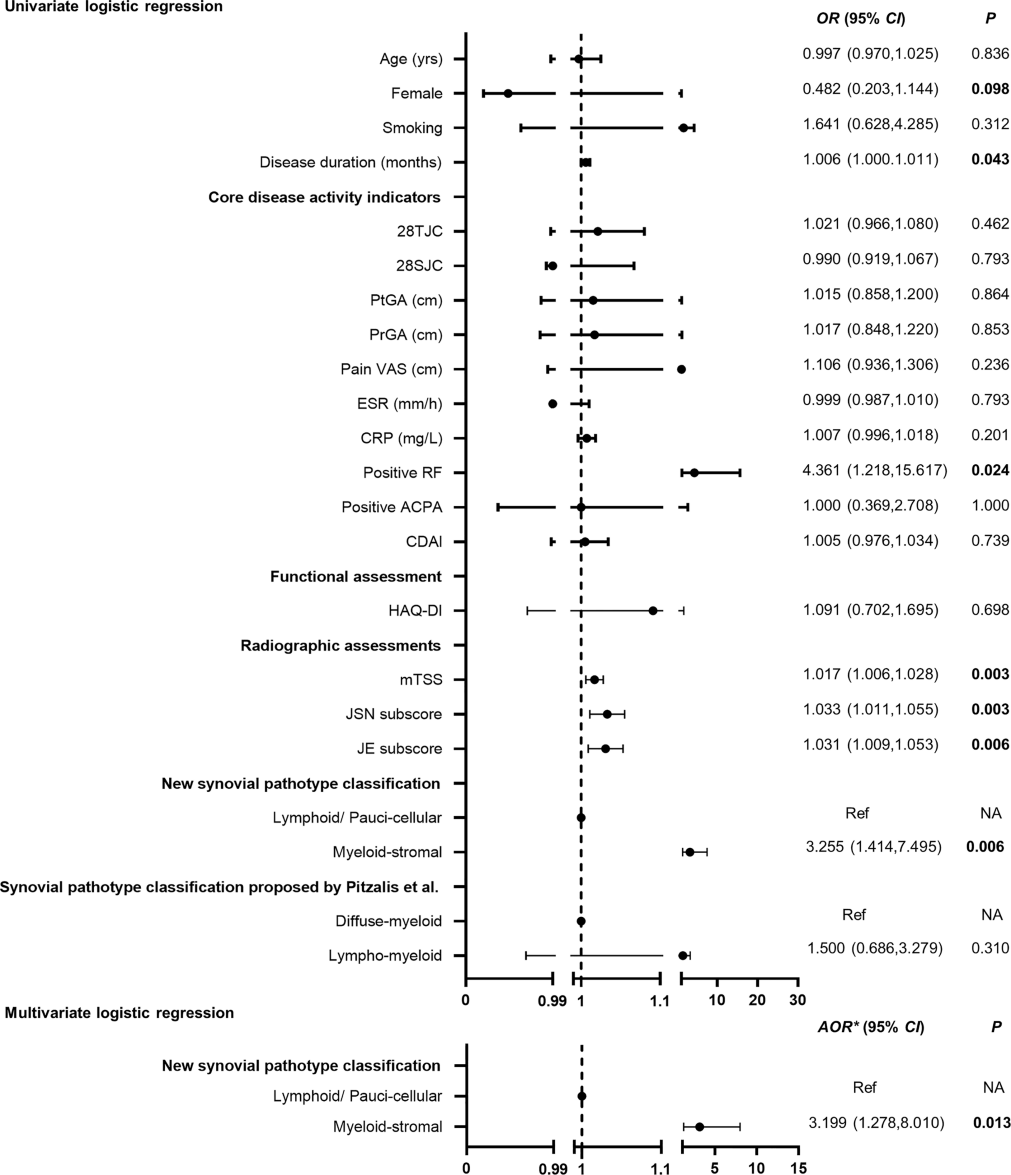
Performance of baseline myeloid-stromal pathotype as a predictor for 1-year radiographic progression. OR, odds ratio; 95% CI, 95% confidence interval; Ref, reference. AOR* adjusted for variables with *p* < 0.1 in the univariate analysis (sex, disease duration, RF status, mTSS) at baseline. NA, data not applicable.

RA patients with baseline myeloid-stromal pathotype showed the highest rate of 1-year radiographic progression versus the lymphoid or paucicellular pathotypes (43.7% vs. 23.1% vs. 7.7%, *p* = 0.011, **Figure 3D**). Univariate logistic regression analysis indicated that myeloid-stromal pathotype at baseline was a significant predictive factor of radiographic progression at 1 year (OR = 3.255, 95% CI: 1.414~7.495, *p* = 0.006, **Figure 4**). Adjusted for confounding factors with *p* < 0.1 in univariate analysis including sex, disease duration, RF status, and mTSS at baseline, the synovial myeloid-stromal pathotype at baseline independently predicted 1-year radiographic progression (AOR = 3.199, 95% CI: 1.278~8.010, *p* = 0.013). Furthermore, five of eight RA patients with RRP showed myeloid-stromal pathotype.

### Subgroup Analysis of New Synovial Pathotypes in Treatment-Naive RA Patients

There were 49 patients who had not received corticosteroid or DMARD treatment for at least 6 months before recruitment. According to our new synovial pathotype classification, 51.0% (25/49) of RA samples were classified as synovial myeloid-stromal pathotype, 36.7% (18/49) as lymphoid pathotype, and 12.2% (6/49) as paucicellular pathotype, which showed similar distribution to the total RA cohort. There were no significant difference in baseline characteristics among RA patients with three synovial pathotypes (all *p* > 0.05, **Supplementary Table S2**). During 1-year follow-up, RA patients with baseline myeloid-stromal pathotype showed the poorest therapeutic response with the lowest rate of Boolean remission (16.0% vs. 50.0% vs. 66.7%, *p* = 0.008, **Table 4**) and CDAI remission (12.0% vs. 50.0% vs. 66.7%, *p* = 0.003), as well as the highest rate of 1-year radiographic progression versus the lymphoid or paucicellular pathotypes (40.0% vs. 5.6% vs. 0%, *p* = 0.011) at 12 months.

**Table 4:** Clinical outcomes in treatment-naive RA patients with different synovial pathotypes during a 1-year follow-up.

Clinical outcomes	New synovial pathotype classification			
	Myeloid-stromal (*n* = 25)	Lymphoid (*n* = 18)	Paucicellular (*n* = 6)	*p* ^a^
**1 month**				
Boolean remission [*n* (%)]	2 (8.0)	3 (16.7)	1 (16.7)	0.576
CDAI remission [*n* (%)]	2 (8.0)	3 (16.7)	1 (16.7)	0.576
CDAI LDA [*n* (%)]	8 (32.0)	9 (50.0)	5 (83.3)	0.082
**3 months**				
Boolean remission [*n* (%)]	6 (24.0)	8 (44.4)	2 (33.3)	0.115
CDAI remission [*n* (%)]	6 (24.0)	8 (44.4)	3 (50.0)	0.300
CDAI LDA [*n* (%)]	16 (64.0)	14 (77.8)	5 (83.3)	0.600
**6 months**				
Boolean remission [*n* (%)]	5 (20.0)	8 (44.4)	2 (33.3)	0.259
CDAI remission [*n* (%)]	5 (20.0)	8 (44.4)	4 (66.7)	0.051
CDAI LDA [*n* (%)]	16 (64.0)	14 (77.8)	5 (83.3)	0.600
**12 months**				
Boolean remission [*n* (%)]	4 (16.0)	9 (50.0)	4 (66.7)	0.008
CDAI remission [*n* (%)]	3 (12.0)	9 (50.0)	4 (66.7)	0.003
CDAI LDA [*n* (%)]	13 (52.0)	14 (77.8)	5 (83.3)	0.177
Radiographic progression in mTSS (*n* (%))	10 (40.0)	1 (5.6)	0 (0)	0.011
Radiographic progression in JSN (*n* (%))	7 (28.0)	1 (5.6)	0 (0)	0.109
Radiographic progression in JE (*n* (%))	7 (28.0)	1 (5.6)	0 (0)	0.109

a Comparisons of baseline characteristics among three new synovial pathotypes by the Chi-square test or Fisher’s exact test. Progression in JSN or JE, defined as a minimum increase of 0 unit in the JSN or JE subscore from baseline to 1 year.

## Discussion

In this real-world study of a prospective RA cohort, we established a new fibroblast-based classification of synovial pathotypes and found that RA patients with baseline myeloid-stromal pathotype had almost 3.2-fold risk on 1-year radiographic progression than those with lymphoid and paucicellular pathotypes. Similar results were obtained for treatment response. Thus, baseline evaluation of synovial pathotypes including stromal cells can provide additional valuable information on prognosis and therapeutic response prediction.

It has been a great challenge to determine if synovial analysis could contribute not only to identify new therapeutic targets but even more be useful to optimize individual therapy in clinical practice (35). The well-accepted and widely used Kenn synovitis score with focus on synovitis severity assessment may be helpful for the diagnosis of RA but has limited association with clinical indicators (14). Recently, Pitzalis et al. proposed three synovial pathotypes using immunohistochemistry results that were associated with molecular signatures identified by RNA-seq data. This classification is highly focused on the degree of infiltration by immunocytes (including lymphocytes, especially B cells, and macrophages) in the sublining synovium (17). B cells and plasma cells are critical for the initiation and maintenance of inflammation, and synovial CD20+ B cells are associated with structural damage progression in very early RA (36, 37). Regarding cells from the monocyte lineage, accumulation of SL-CD68+ macrophages was associated with more severe disease activity and radiographic progression and to treatment response (38, 39). In line with these previous studies, this study showed that the Krenn synovitis score showed no association with changes of disease activity or radiographic progression. However, higher amounts of synovial CD20+ B cells and of SL-CD68+ macrophages at baseline were associated with persistent disease activity and increased radiographic progression at 1 year.

Apart from immunocytes, activation of stromal cells namely endothelial cells and fibroblasts, also participates in RA synovitis (16, 40). The question is thus if adding markers of these cells would improve the value of synovial pathotypes? Increased density of endothelial cells perpetuates synovitis and allows immunocytes to migrate from blood into inflamed synovium. The recruited immunocytes then produce proinflammatory cytokines and chemokines leading to joint inflammation and destruction (20). Synovial fibroblasts show high heterogeneity in RA and have unique aggressive behaviors by producing various cytokines, chemokines, and matrix-degrading enzymes that prompt disease initiation and perpetuation (41). Recently, a study showed that CD34-CD90+ fibroblasts are associated with increased gene expression related to osteoclastogenesis by single-cell analysis of RA synovium (25). High expression of PDPN on RA synoviocytes was linked with upregulated IL-17 production in a coculture system of peripheral blood mononuclear cells (PBMC) and synoviocytes, implying the critical role of PDPN in the high IL-17 secretion during cell interactions (23). Fibroblast activation protein-α (FAPα) colocalized with PDPN in both lining and sublining cells, and FAP may be associated with a pathogenic fibroblast phenotype, contributing to increased cartilage degradation and synovial inflammation (42). A recent study showed that injection of PDPN+FAPα+CD90+ cells into the inflamed ankle joint of mice resulted in more severe and sustained joint swelling with increased leukocyte infiltration. Injection of PDPN+FAPα+CD90− cells results in increased structural joint damage, implying that CD90 may be another biomarker of sustained tissue inflammation (43). Another recent study proposed a new role of endothelium-derived Notch3 signaling pathway on the positional identification and expansion of perivascular CD90+ fibroblasts, leading to an acceleration of synovitis and articular damage in mice (44). Considering the important roles of CD90+ fibroblasts and CD31+ endothelial cells in RA and difficulties in distinguishing endothelial cells and perivascular fibroblasts by H&E staining, we added these two cell markers to the immunologic synovitis assessment. Our results showed that higher amounts of CD31+ endothelial cells at baseline were associated with cumulative joint destruction at 1 year. Higher amounts of CD90+ fibroblasts at baseline were significantly associated with persistent disease activity during 1-year follow-up, which indicated the importance of stromal cells in the immunologic synovitis assessment.

The use of genomics has extended our understanding of RA synovial tissue heterogeneity. Three distinct synovial subtypes defined by gene expression cluster analysis of RA synovium have been identified including a high inflammatory subtype associated with high gene expression of inflammation markers and autoantibodies leading to higher disease activity, a low inflammatory subtype associated with high neuronal and glycoprotein gene expression and a mixed subtype with features with both the high and low inflammatory subtypes (45, 46). Another study divided RA synovium into lymphoid, myeloid, low inflammatory and fibroid phenotypes, and patients with myeloid phenotype showed good therapeutic response to the anti-TNFα treatment. This study also identified the value of high baseline serum soluble intercellular adhesion molecule 1 (sICAM1) and C-X-C motif chemokine 13 (CXCL13), associated with the myeloid and lymphoid phenotype, respectively. RA patients with high sICAM1 and low CXCL13 showed the highest ACR50 response under anti-TNF-α treatment after 24 weeks. This work provides new directions to identify possible serum biomarkers associated with the different synovial pathotypes (47). In the studies of Pitzalis et al., RA patients with lymphomyeloid pathotype showed more severe disease activity and cumulative joint destruction in early and treatment-naïve RA, but no significant difference was found in the response to DMARD treatment among different pathotypes (17). RA patients with lymphomyeloid and diffuse-myeloid pathotype showed greater DAS28 improvement than patients with pauci-immune pathotypes only under anti-TNF treatment (48). In our real-world cohort, according to our synovial pathotype classification that replaced the CD3 and CD38 markers by CD31 and CD90, to give more priority to myeloid and stromal cells, RA patients could be stratified into different subgroups with different risk ratios of clinical outcomes, especially 1-year radiographic progression. Considering that synovial composition could be changed by RA treatment, along with a shift of synovial pathotypes (48, 49), the subgroup analysis in treatment-naive RA patients were performed and confirmed the results that were obtained in the whole cohort. These results indicated the additional value of the stromal cell markers to the synovial immunohistological classification.

This study has several limitations. First, our study used a single synovial biopsy in active RA patients without patients in remission. Studies have shown that the synovial pathotypes can shift during different disease stages in the same patient, possibly through different steps of joint inflammation and chronicity (16, 49). The application of our proposed synovial pathotypes to RA in remission although more complex to perform would be of interest. Second, in our study, radiographic progression was based on hand X-rays whereas synovial pathotypes were obtained from knee joints. A recent study showed a higher proportion of pauci-immune pathotype characterized by fibroblast expansion in small- and medium-sized joints compared with knees (17). Thus, the cellular and molecular profiles of synovium may vary from different inflammatory joints in each individual patient (16). The application of our proposed synovial pathotypes to hands and other joints is worth of further investigation. Third, most patients had a long disease duration and received different treatments before recruitment in this single-center study. Although the results obtained in the whole cohort were confirmed in the subgroup of treatment-naive RA, they need to be extended to patients with early and treatment-naïve disease in multicenter trials. Single-cell technology has identified various cell surface molecules to identify different fibroblast subsets, including cadherin-11, CD55, PDPN, FAP, CD90, and vimentin (50). The clinical significance of these different fibroblast subsets identified by markers other than CD90 needs further investigation. Additionally, although the semiquantitative analysis in our study was valid and mirrored previous works done by Pitzalis and colleagues (17, 48), further artificial intelligence analysis would be adopted to test the validity of this method.

In conclusion, our study is the first to introduce stromal cells into a classification of synovial pathotypes in RA. Addition of stromal cell markers to a novel synovial myeloid-stromal pathotype improved the predictive value of the classification. It could predict radiographic progression and poor therapeutic response at 1 year. Addition of the sublining stromal cells may extend the power of existing immunocyte-based synovial pathotype. Application of this new synovial pathotype classification in the kinetic studies during clinical trials may contribute to a shift of therapeutic decisions for personalized therapy in RA.

## Data Availability Statement

The original contributions presented in the study are included in the article/**Supplementary Material**. Further inquiries can be directed to the corresponding authors.

## Ethics Statement

The studies involving human participants were reviewed and approved by the Medical Ethics Committee of Sun Yat-Sen Memorial Hospital (SYSEC-2009-06 and SYSEC-KY-KS-012). The patients/participants provided their written informed consent to participate in this study.

## Author Contributions

X-PZ, J-DM, and Y-QM contributed equally to this work, including conceiving and designing the study, reading and analyzing documents, performing the statistical analysis, and drafting the manuscript. Corresponding authors PM and LD also conceived and participated in its design, advised on the search, read and analyzed documents, and edited the paper. JJ, D-HZ, and L-FC carried out needle synovial biopsy and critically revised the manuscript. C-TC and QZ participated in clinical assessments of RA patients and data analysis. X-PZ and TW carried out immunohistochemical staining and histopathological assessments. Y-YZ and J-ZL advised on the search and critically revised the manuscript. J-DM and Z-HY carried out the radiographic assessment. Y-HX and Y-WZ participated in data collection and critically revised the manuscript. LL advised on the study design and statistical analysis. All authors contributed to the article and approved the submitted version.

## Funding

This work was supported by the National Natural Science Foundation of China (81801606 to J-DM, 81971527 to LD, and 82001731 to JJ),Natural Science Foundation of Guangdong Province (2018A030313541 to J-DM and 2018A030313690 to Y-YZ), Guangdong Medical research Foundation (A2018062 to Y-YZ and A2021065 to J-ZL), Guangzhou Municipal Science and Technology Project (201904010088 to LD and 202102010188 to J-DM), and Guangdong Basic and Applied Basic research Foundation (2019A1515011928 to LD, 2019A1515110125 to JJ, and 2020A1515110061 to J-ZL).

## Conflict of Interest

The authors declare that the research was conducted in the absence of any commercial or financial relationships that could be construed as a potential conflict of interest.

## Publisher’s Note

All claims expressed in this article are solely those of the authors and do not necessarily represent those of their affiliated organizations, or those of the publisher, the editors and the reviewers. Any product that may be evaluated in this article, or claim that may be made by its manufacturer, is not guaranteed or endorsed by the publisher.

## Supplementary Material

Supplementary Figure 1 Dynamic changes in clinical indicators and therapeutic responses of RA patients during one-year follow-up. **(A–J)** Dynamic changes in disease activity indicators including 28TJC, 28SJC, PtGA, PrGA, Pain VAS, ESR, CRP, DAS28-CRP, SDAI and CDAI. **(K)** Dynamic changes in HAQ-DI. **(L–N)** Dynamic changes in therapeutic responses including rates of Boolean remission, CDAI remission and CDAI LDA.

Supplementary Figure 2 Comparisons of dynamic changes in clinical indicators during one-year follow-up among RA patients with different synovial pathotypes in the new synovial pathotype classification. **(A–J)** Comparisons of dynamic changes in disease activity indicators including 28TJC, 28SJC, PtGA, PrGA, Pain VAS, ESR, CRP, DAS28-CRP, SDAI and CDAI. **(K)** Comparisons of dynamic changes in HAQ-DI. **P*<0.05, ***P*<0.01 (Kruskal-Wallis test).
