# Supplementary figures and images for: Addition of Fibroblast-Stromal Cell Markers to Immune Synovium Pathotypes Better Predicts Radiographic Progression at 1 Year in Active Rheumatoid Arthritis
Source: Front Immunol. 2021 Nov 23;12:778480. doi: 10.3389/fimmu.2021.778480 (PMC8650215; doi:10.3389/fimmu.2021.778480)

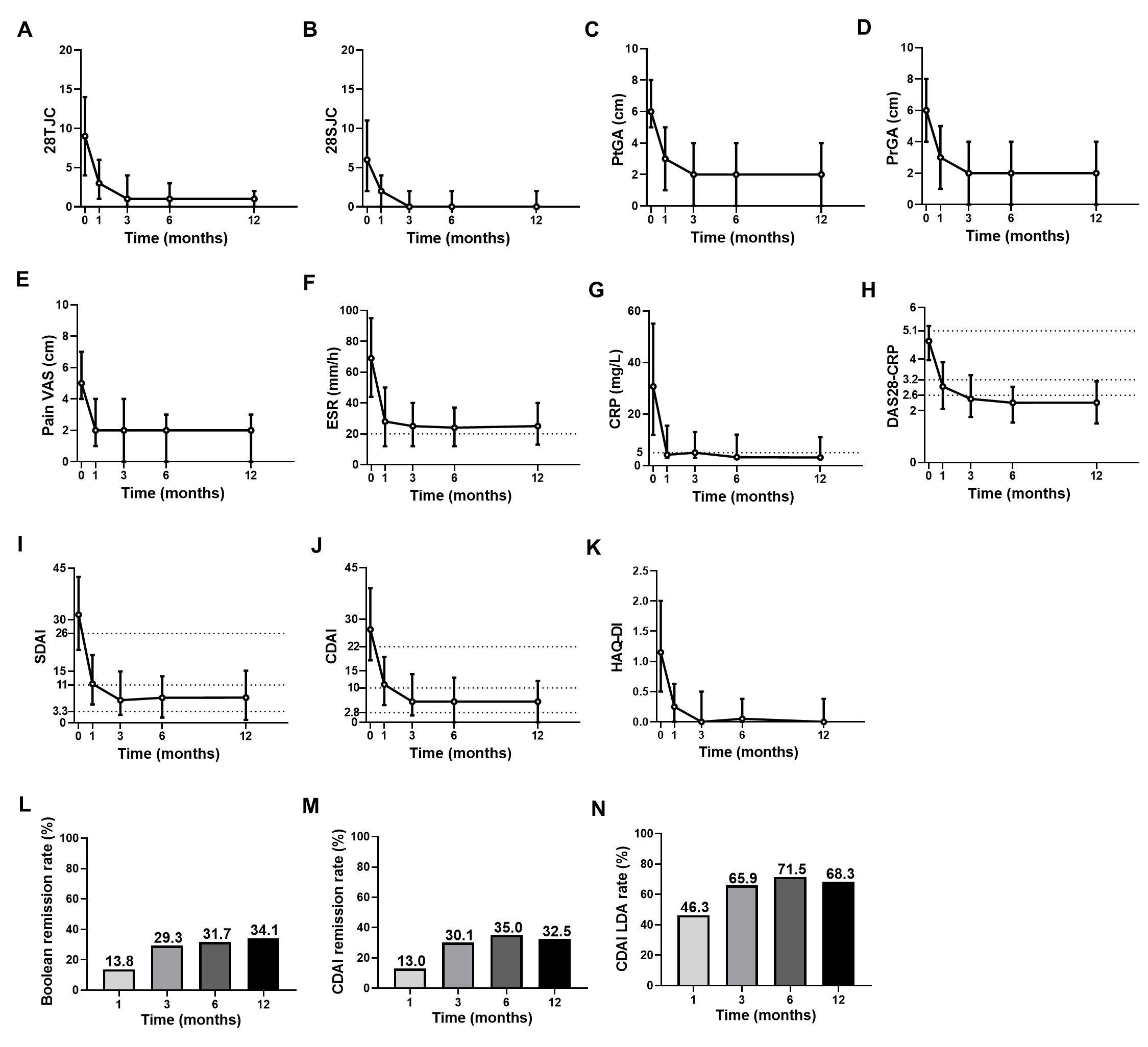

Supplement: Supplementary Figure 1 — Dynamic changes in clinical indicators and therapeutic responses of RA patients during one-year follow-up. (A–J) Dynamic changes in disease activity indicators including 28TJC, 28SJC, PtGA, PrGA, Pain VAS, ESR, CRP, DAS28-CRP, SDAI and CDAI. (K) Dynamic changes in HAQ-DI. (L–N) Dynamic changes in therapeutic responses including rates of Boolean remission, CDAI remission and CDAI LDA. [file Image_1.tif]

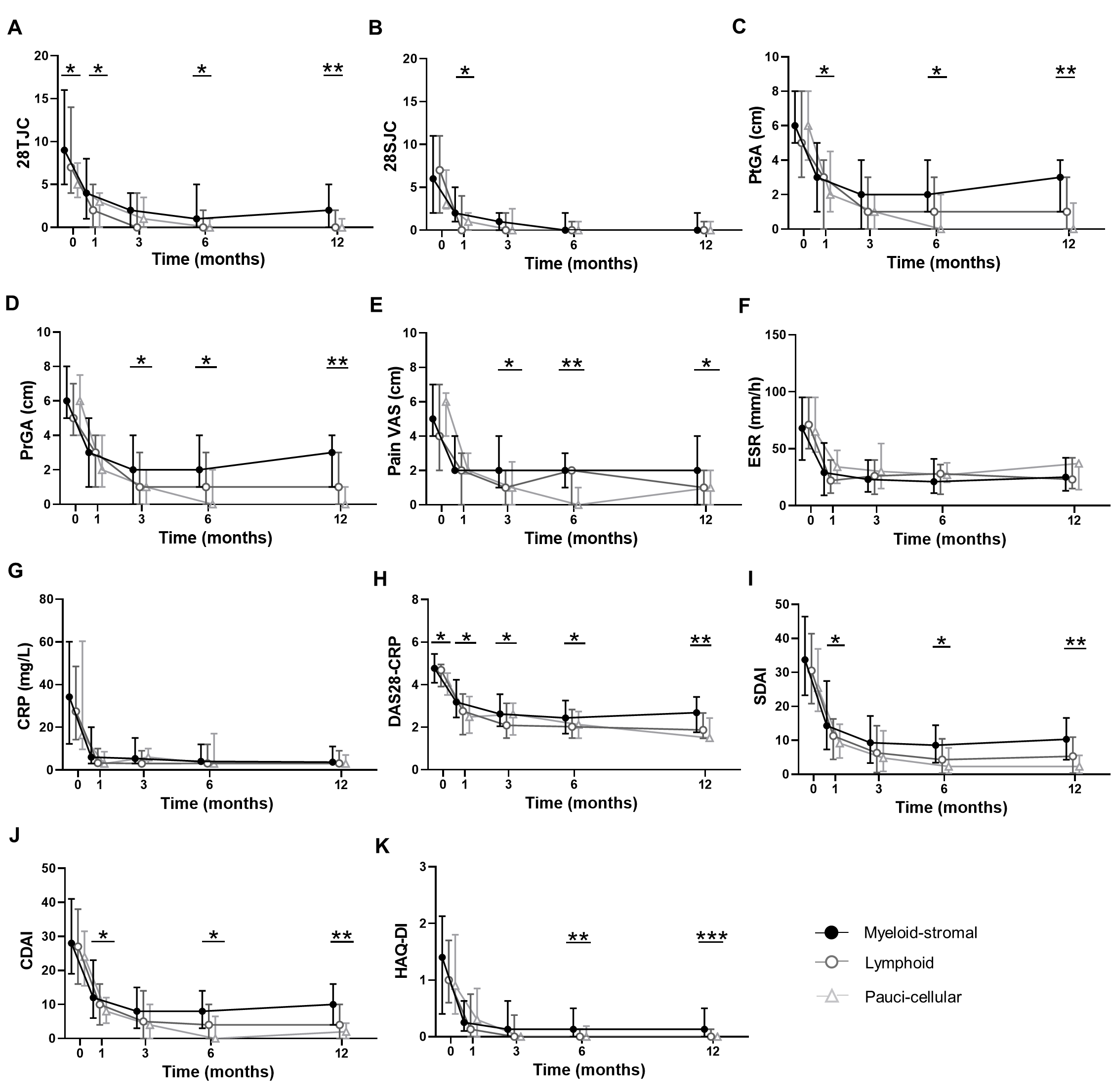

Supplement: Supplementary Figure 2 — Comparisons of dynamic changes in clinical indicators during one-year follow-up among RA patients with different synovial pathotypes in the new synovial pathotype classification. (A–J) Comparisons of dynamic changes in disease activity indicators including 28TJC, 28SJC, PtGA, PrGA, Pain VAS, ESR, CRP, DAS28-CRP, SDAI and CDAI. (K) Comparisons of dynamic changes in HAQ-DI. *P<0.05, **P<0.01 (Kruskal-Wallis test). [file Image_2.tif]
