# Supplementary material for: Addition of Fibroblast-Stromal Cell Markers to Immune Synovium Pathotypes Better Predicts Radiographic Progression at 1 Year in Active Rheumatoid Arthritis
Source: Front Immunol. 2021 Nov 23;12:778480. doi: 10.3389/fimmu.2021.778480 (PMC8650215; doi:10.3389/fimmu.2021.778480)
Supplement: Supplementary file 3 [file Table_1.docx]

**Supplementary materials**

**Supplementary Tables**

**Table S1 Baseline characteristics in all RA patients with different synovial pathotypes**

|  | New synovial pathotype classification | | | |
| --- | --- | --- | --- | --- |
| Characteristics | Myeloid-stromal (n=71) | Lymphoid  (n=39) | Pauci-cellular  (n=13) | *P^*^* |
| Age, yr., mean ± SD | 51±15 | 49±13 | 48±11 | 0.675 |
| Female, n (%) | 56 (78.9) | 31 (79.5) | 8 (61.5) | 0.360 |
| Disease duration, months, mean ± SD | 71±82 | 44±54 | 34±60 | 0.083 |
| Smoking, n (%) | 13 (18.3) | 5 (12.8) | 3 (23.1) | 0.636 |
| Core disease activity indicators |  |  |  |  |
| TJC28, median (IQR) | 9 (5,16) | 7 (4,14) | 5 (4,8) | 0.023 |
| SJC28, median (IQR) | 6 (2,11) | 7 (2,11) | 3 (3,7) | 0.417 |
| PtGA, median (IQR) | 6 (5,8) | 5 (3,8) | 6 (4,8) | 0.548 |
| PrGA, median (IQR) | 6 (5,8) | 5 (4,7) | 6 (4,8) | 0.311 |
| Pain VAS, median (IQR) | 5 (4,7) | 4 (2,7) | 6 (4,7) | 0.537 |
| ESR (mm/h), median (IQR) | 68 (40,95) | 71 (50,95) | 65 (47,95) | 0.758 |
| CRP (mg/L), median (IQR) | 34.2 (12.2,60.1) | 27.4 (14.2,48.5) | 16.4 (9.7,60.3) | 0.647 |
| Positive RF, *n* (%) | 55 (77.5) | 32 (82.1) | 12 (92.3) | 0.443 |
| Positive ACPA, *n* (%) | 57 (80.3) | 32 (82.1) | 13 (100.0) | 0.218 |
| DAS28-CRP, median (IQR) | 4.8 (4.1,5.4) | 4.7 (3.9,5.0) | 4.1 (3.5,4.5) | 0.038 |
| SDAI, median (IQR) | 33.7 (23.3,46.4) | 30.5 (20.8,41.4) | 25.5 (18.6,36.9) | 0.152 |
| CDAI, median (IQR) | 28 (19,41) | 27 (16,38) | 24 (16,32) | 0.153 |
| Functional indicator |  |  |  |  |
| HAQ-DI, median (IQR) | 1.4 (0.4,2.1) | 1.0 (0.6,1.7) | 0.9 (0.4,1.8) | 0.451 |
| Radiographic assessments |  |  |  |  |
| mTSS, median (IQR) | 14 (4,49) | 8 (2,24) | 3 (0,6) | 0.001 |
| JSN subscore, median (IQR) | 7 (2,24) | 2 (0,14) | 1 (0,2) | 0.002 |
| JE subscore, median (IQR) | 10 (1,23) | 5 (0,12) | 2 (0,5) | 0.004 |
| Previous medications |  |  |  |  |
| Treatment naive^Δ^, *n* (%) | 25 (35.2) | 18 (46.2) | 6 (46.2) | 0.472 |
| Corticosteroids, *n* (%) | 35 (49.3) | 19 (48.7) | 5 (38.5) | 0.767 |
| csDMARDs, n (%) | 26 (36.6) | 18 (41.0) | 7 (69.2) | 0.090 |
| Biologic agents, *n* (%) | 7 (9.9) | 6 (15.4) | 0 (0) | 0.385 |

^*^Comparisons of baseline characteristics among three new synovial pathotypes by the Chi-square test or Fisher exact test for qualitative variables, ANOVA or Kruskal-Wallis test for quantitative variables.

**Table S2 Baseline characteristics in treatment-naive RA patients with different synovial pathotypes**

|  | **New synovial pathotype classification** | | | |
| --- | --- | --- | --- | --- |
| **Characteristics** | Myeloid-stromal (n=25) | Lymphoid  (n=18) | Pauci-cellular  （n=6） | *P^*^* |
| Age, yr., mean ± SD | 52 ± 11 | 50 ± 14 | 48 ± 14 | 0.921 |
| Female, n (%) | 20 (80.0) | 16 (88.9) | 3 (50.0) | 0.134 |
| Disease duration, months, mean ± SD | 65 ± 63 | 41 ± 62 | 54 ± 82 | 0.335 |
| Smoking, n (%) | 5 (20.0) | 2 (11.1) | 2 (33.3) | 0.456 |
| **Core disease activity indicators** |  |  |  |  |
| TJC28, median (IQR) | 12 (5,16) | 10 (5,15) | 5 (4,7) | 0.125 |
| SJC28, median (IQR) | 6 (3,11) | 7 (3,12) | 3 (2,7) | 0.415 |
| PtGA, median (IQR) | 6 (5,8) | 6 (3,7) | 7 (5,9) | 0.745 |
| PrGA, median (IQR) | 6 (5,8) | 5 (5,7) | 7 (5,9) | 0.485 |
| Pain VAS, median (IQR) | 5.0 (4.0,6.0) | 4.5 (3.8,7.3) | 6.5 (3.5,7.3) | 0.691 |
| ESR (mm/h), median (IQR) | 59.0 (37.5,89.5) | 70.5 (53.0,97.8) | 66.5 (50.5,96.0) | 0.227 |
| CRP (mg/L), median (IQR) | 23.0 (9.1,54.7) | 24.2 (10.7,49.8) | 15.4 (8.6,50.2) | 0.900 |
| Positive RF, *n* (%) | 18 (72.0) | 15 (83.3) | 6 (100.0) | 0.322 |
| Positive ACPA, *n* (%) | 20 (80.0) | 16 (88.9) | 6 (100.0) | 0.614 |
| DAS28-CRP, median (IQR) | 4.8 (4.1,5.4) | 4.6 (4.0,5.0) | 4.1 (3.5,4.2) | 0.085 |
| SDAI, median (IQR) | 35.0 (23.3,44.7) | 30.4 (21.1,43.0) | 25.1 (18.5,31.9) | 0.327 |
| CDAI, median (IQR) | 31.0 (18.5,40.0) | 28.0 (18.5,41.3) | 20.0 (16.8,30.3) | 0.515 |
| **Functional indicator** |  |  |  |  |
| HAQ-DI, median (IQR) | 1.4 (0.4,2.1) | 1.1 (0.7,2.1) | 1.1 (0.8,2.1) | 0.983 |
| **Radiographic assessments** |  |  |  |  |
| mTSS, median (IQR) | 12 (2,51) | 8 (3,11) | 6 (1,12) | 0.445 |
| JSN subscore, median (IQR) | 5 (0,25) | 1 (0,5) | 2 (1,7) | 0.184 |
| JE subscore, median (IQR) | 12 (1,24) | 5 (1,10) | 4 (1,5) | 0.369 |

^*^Comparisons of baseline characteristics among three new synovial pathotypes by the Chi-square test or Fisher exact test for qualitative variables, ANOVA or Kruskal-Wallis test for quantitative variables.
